# Efficacy of direct anterior approach combined with direct posterior approach in Pipkin IV femoral head fractures

**DOI:** 10.1186/s13018-022-03058-9

**Published:** 2022-03-12

**Authors:** Bo Liu, Binghao Zhao, Qingsong Zhang

**Affiliations:** Department of Osteoarthrosis, Renmin Hospital, Hubei University of Medicine, No.39 Middle Chaoyang Road, Maojian District, Shiyan, 442000 Hubei China

**Keywords:** Direct anterior approach, Direct posterior approach, Pipkin IV femoral head fractures, Efficacy of application

## Abstract

**Objective:**

The study aimed to explore the efficacy of direct anterior approach combined with direct posterior approach in Pipkin IV femoral head fractures.

**Methods:**

The study enrolled 64 patients with Pipkin IV femoral head fractures who were treated at our hospital between March 2019 and April 2020. They were assigned to the control group and the study group using the random number table method with 32 patients in each group and received treatment by the direct anterior approach and treatment by the direct anterior approach combined with the direct posterior approach. The operative time, intraoperative estimated blood loss, postoperative drainage time, drainage volume, time to partial and full weight-bearing, total length of hospital stay and the levels of hemoglobin (Hb) and hematocrit (Hct) in the two groups were compared, and severity of pain and hip function at different time points postoperatively were observed, and the occurrences of complications were compared.

**Results:**

There was no statistical difference in the operative time and intraoperative estimated blood loss between the two groups (*P* > 0.05). Compared with the control group, the study group had shorter postoperative drainage time, lower drainage volume, shorter time to partial and full weight-bearing, and shorter total length of hospital stay, and the difference was statistically different (*P* < 0.05). There was no significant difference in Hb and Hct levels between the two groups before surgery (*P* > 0.05). The levels of Hb and Hct in both groups at postoperative day (POD) 1 were lower than those before surgery, and the levels of Hb and Hct in the study group were significantly higher than those in the control group (*P* < 0.05). Compared with the control group, the study group had significantly less severe pain at POD 1 and 7 and 1, 3 and 6 months postoperatively (*P* < 0.05). Compared with the control group, the study group had significantly better hip function at 3, 6 and 12 months postoperatively (*P* < 0.05). All patients were followed up for 12 months, and 1 case of ectopic ossification appeared in both groups 3 months postoperatively, both Brooker grade I. No special treatment was provided as it did not interfere with the mobility of the hip and caused no apparent discomfort in the patients. In the current study, no incision infection, ischemic necrosis of the femoral head, breakage of the internal fixation device, fracture nonunion and loss of fracture reduction and other complications were reported in any patients.

**Conclusion:**

Direct anterior approach combined with direct posterior approach in Pipkin IV femoral head fractures does not increase operative time and intraoperative estimated blood loss but can lessen severity of pain and promote functional recovery of the hip, leading to a favorable prognosis while not increasing the incidence of complications.

## Introduction

Over the recent years, with continuing development of the transport industry, the number of traffic accidents has been gradually on the rise, and the incidence of femoral head fractures has also steadily climbed [1]. Pipkin fracture, which is posterior dislocation of the hip combined with femoral head fractures, is mainly caused by high energy injuries [2]. Based on Pipkin fracture classification, it can be categorized into four types; the incidence of Pipkin IV fracture is about 27% [2]. If Pipkin IV fracture does not receive prompt and effective treatment, complications such as ischemic necrosis of the femoral head, traumatic arthritis of the hip and ectopic ossification could occur, which greatly impact on daily life activities of patients and their work. Therefore, it remains an urgent and clinically important issue how to effectively restore the normal anatomy and morphology of the hip and reduce the occurrence of complications while aiming at achieving good hip function in the patients.

Currently, surgical treatment is undertaken for Pipkin IV femoral head fractures, and common surgical approaches include direct anterior approach and Kocher-Langenbeck (K–L) approach [3, 4]. However, the surgical protocol for Pipkin IV femoral head fractures should not just take into consideration the femoral head and acetabulum because femoral head fracture fragments are often located in the anteromedial femoral head, and among them, posterior wall acetabular fractures are the most common type of acetabulum fractures. Because a single surgical incision cannot fully expose the anterior and posterior aspect of the hip, it has certain limitations [5]. Currently, the surgical approach for Pipkin IV femoral head fractures remains controversial. Hence, the current study aimed to explore the efficacy of direct anterior approach combined with direct posterior approach in Pipkin IV femoral head fractures.

## Patients and methods

### Clinical data

The study enrolled 64 patients with Pipkin IV femoral head fractures who were treated at our hospital between March 2019 and April 2020. They were assigned to the control group and the study group with 32 patients in each group using the random number table method. In the study group, the age ranged from 23 to 54 years, with a mean age of (43.01 ± 9.12) years; there were 18 males and 14 females. The fracture was on the left in 18 cases and on the right in 14 cases. The causes of injury were traffic accidents in 28 cases and injury due to fall from height in 4 cases. In the control group, the age ranged from 22 to 53 years, with a mean age of (42.91 ± 9.09) years. There were 19 males and 13 females. The fracture was on the left in 19 cases and on the right in 13 cases. The causes of injury were traffic accidents in 29 cases and injury due to fall from height in 3 cases.

The study protocol followed the Declaration of HELSINKI of the World Medical Association.

### Eligibility criteria

The inclusion criteria were as follows: Pipkin IV femoral head fractures; posterior wall acetabular fracture combined with femoral head fractures according to preoperative radiograph and CT scan; marked displacement of fracture; complete clinical data; the time from injury to operation was < 2 weeks. The exclusion criteria were as follows: concurrent femoral neck fracture; open fracture; a history of previous hip diseases; coagulation abnormalities; follow up duration < 3 months or lost to follow up; concurrent severe brain or internal organ injury, severe lower extremity injury; severe osteoporosis; concurrent bone tumor; concurrent autoimmune diseases. We also excluded patients who cannot tolerate surgery, or who had poor compliance. Mentally abnormal patients were excluded as well.

### Methods

#### Preoperative preparation

All patients underwent closed reduction of hip dislocation after admission, followed by traction via the femoral condyle or the tibial tubercle. Analgesics and anticoagulation therapy were given simultaneously. The anteroposterior and Judet view of the pelvis was taken, and pelvic CT and preoperative tests (liver and renal function and blood routines) were done. Surgical treatment was done 2–11 days post-injury. The patients were fasted for 8 h before surgery, and broad-spectrum antibiotics were given 30 min before surgery. All patients were operated by the same group of similarly experienced surgeons.

#### Direct anterior approach

**Fig. 1:**
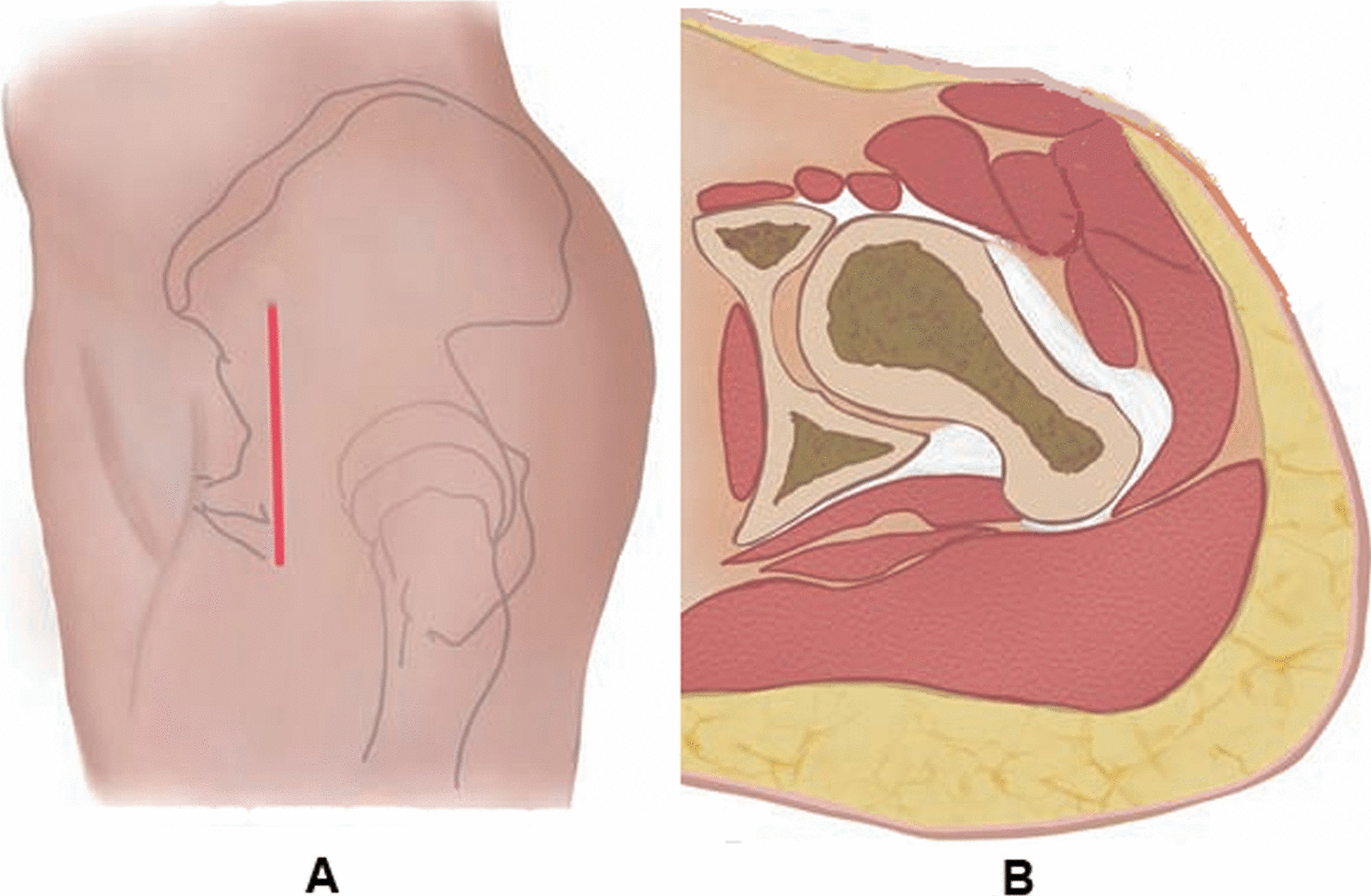
Schematic diagram of surgical incision through the direct anterior approach. **A** Longitudinal incision is made caudally about 2-cm-lateral to the distal anterior superior iliac spine along the direction of the tensor fascia lata. **B** The tensor fascia lata is pulled laterally, and the inner fascia, the sartorius, and rectus femoris are pulled medially to fully expose the femoral neck

After general anesthesia, the patient was placed in the supine position, and a longitudinal incision was made about 2-cm-lateral to the distal anterior superior iliac spine in the direction of the tensor fascia lata downward (Fig. 1A). After the superficial fascia of the tensor fascia lata was incised, the muscle layer of the tensor fascia lata was dissected bluntly from the interior fascia along the fatty band, the tensor fascia lata was pulled laterally, and the inner fascia, the sartorius and rectus femoris were pulled medially to fully expose the femoral neck (Fig. 1B). The lateral femoral artery was clearly visible in the surgical field, and the lateral femoral circumflex artery, if necessary, was ligated with a knot. The articular capsule and the rectus femoris reflected head were fully exposed and then separated. The articular capsule was incised in a Z form, and the affected limb was placed under traction. After the femoral head fracture was fully exposed, the soft tissues and hematoma in the broken end of the fracture were debrided, and the femoral head fracture fragments underwent anatomic reduction under direct vision. Countersunk lag screws (Synthes, Swiss) were used for stabilization. Fracture reduction and screw position were observed under C**-**arm fluoroscopy and if satisfactory, the wound was irrigated**,** the articular capsule was repaired. After placement of the drainage tube, the incision was sutured layer by layer. The schematic diagram of surgical incision via the direct anterior approach is shown in Fig. 1.

#### Direct posterior approach

**Fig. 2:**
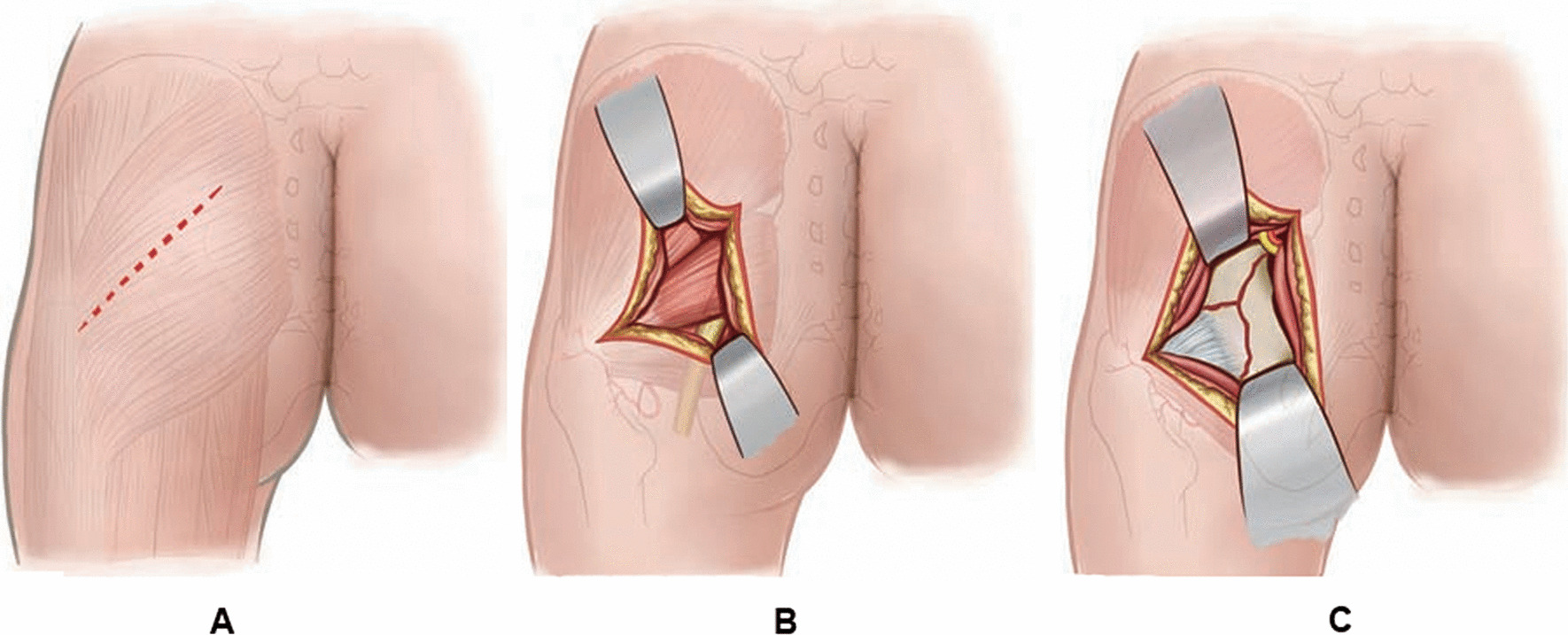
Schematic diagram of surgical incision through direct posterior approach. **A** A straight incision is made from the midpoint of the line drawn between the posterior border of the tip of the greater trochanter and the posterior superior iliac spine to the posterior border of the greater trochanter. **B** The gluteus maximus is split along the muscle fibers and pulled laterally on both sides. **C** The superior portion of the greater sciatic foramen is exposed, and the superior gluteal vessels and nerves are protected and stripped along the periosteum, and the bone fragment of the posterior wall of the acetabulum is exposed

The posterior superior iliac spine and the posterior border of the tip of the greater trochanter were used as the landmarks for skin incision. A straight incision was made from the midpoint of the line drawn between the posterior border of the tip of the greater trochanter and the posterior superior iliac spine to the posterior border of the greater trochanter (Fig. 2A). The gluteus maximus was split along the muscle fibers and pulled laterally on both sides (Fig. 2B), and the gluteus medius was pulled anterosuperiorly without transecting the abductor muscle and the lateral rotator muscles; the piriformis and other supinator muscles were pulled posteroinferiorly. The superior portion of the greater sciatic foramen was exposed, and the superior gluteal vessels and nerves were protected and stripped along the periosteum. Then, the bone fragment of the posterior wall of the acetabulum was exposed (Fig. 2C) and lifted, and hematoma in the acetabulum was irrigated. After reduction under direct vision, a preset acetabulum arc-shaped reconstruction plate was placed along the rim of the acetabulum to stabilize the fracture, and anchors were used to stabilize smaller posterior wall acetabular fractures. After satisfactory fracture reduction and screw position were confirmed by C**-**arm fluoroscopy, the wound was irrigated, and the incision was sutured layer by layer after drainage tube placement. The schematic diagram of surgical incision via the direct posterior approach is shown in Fig. 2.

#### Postoperative management

Antibiotics were given for 24 h postoperatively to prevent infection. Oral rivaroxaban was prescribed for 1 week to prevent thrombosis; pressure stockings were worn in bilateral lower limbs. The drainage tube was removed when the drainage volume was less than 50 mL/day. Fracture reduction was examined postoperatively by pelvic radiograph and CT 3D reconstruction. The quadriceps muscle of the affected side underwent isometric contraction exercise on postoperative day (POD) 1, and active/inactive activities of the affected limb depended on the condition of the patient 1–2 weeks postoperatively, and fracture reduction, fixation and concurrent injuries were observed, and the patient started assisted partial weight-bearing 6–10 weeks postoperatively and full weight-bearing 12–16 weeks postoperatively.

### Patient assessments

The operative time, intraoperative estimated blood loss, postoperative drainage time and volume, time to partial and full weight-bearing and total length of hospital stay in the two groups were compared. In addition, the severity of pain and hip function at different time points postoperatively were observed. Pain was assessed using the visual analog scale (VAS) [6], with a score of 0–10. Higher scores indicated greater severity of pain. Hip function was evaluated using Harris hip function [7], which assessed range of motion of the joint, deformity, function and pain. The total score ranged from 0 to 100, and higher scores indicated better hip function.

Three to 5 mL fasting blood were collected via the cubital vein before and 24 h after surgery. Hemoglobin (Hb) and hematocrit (Hct) were detected by cell analyzer after anticoagulation treatment.

Complications including ectopic ossification, surgical incision infection, ischemic necrosis of the femoral head, breakage of the internal stabilization device, fracture nonunion and loss of fracture reduction during the 12 months’ follow up were recorded. The rates of complications were compared.

### Statistical analysis

Data were analyzed using SPSS 21.0 software. Quantitative data were expressed in $\overline{x} \pm s$ and examined using Student’s *t* test. Categorical data were expressed as rate (%) and examined using chi-square (*χ*^2^) test. *P* < 0.05 indicated statistically significant difference.

## Results

### Comparison of general data between the two groups

There was no statistical difference in the mean age, location and causes of injury, concurrent injuries, femoral head fracture line and posterior wall acetabular fracture between the two groups (*P* > 0.05) (Table 1).

**Table 1:** Comparison of general data between the two groups

Variables	The study group (32)	The control group (32)
Mean age (years)	43.01 ± 9.12	42.91 ± 9.09
M/F	18/14	19/13
Laterality		
Left/right	18/14	19/13
Causes of injury		
Car accidents	28	29
Injury due to fall from height	4	3
Concurrent injuries		
Patella fracture	1	1
Rib fracture	3	2
Frontal bone fracture	1	2
Head injury	1	1
Femoral head fracture line		
Fracture line below the fossa capitis femoris	7	5
Fracture line above the fossa capitis femoris	3	2
Letournel–Judet classification		
Posterior wall acetabular fracture	8	7
Posterior column and posterior wall acetabular fracture	3	2
Transverse and posterior wall acetabular fracture	2	1

^#^*P* < 0.05 vs. the control group

### Comparison of operative time, intraoperative estimated blood loss, postoperative drainage time and volume, time to partial and full weight-bearing and total length of hospital stay between the two groups

There was no statistical difference in the operative time and intraoperative estimated blood loss between the two groups (*P* > 0.05). Compared with the control group, the study group had a shorter postoperative drainage time, a lower drainage volume, a shorter time to partial and full weight-bearing and a shorter total length of hospital stay, with statistically significant difference between the groups (*P* < 0.05) (Table 2).

**Table 2:** Comparison of operative time, intraoperative estimated blood loss, postoperative drainage time, drainage volume, time to partial and full weight-bearing and total length of hospital stay ($\overline{x} \pm s$)

Variables	The study group (32)	The control group (32)
Operative time (min)	181.93 ± 32.92	194.93 ± 34.91
Intraoperative estimated blood loss (mL)	482.87 ± 78.93	509.87 ± 79.93
Postoperative drainage time (days)	0.78 ± 0.13^#^	1.78 ± 0.18
Postoperative drainage volume (mL)	69.93 ± 12.19^#^	98.83 ± 14.92
Time to partial weight-bearing (days)	1.34 ± 0.31^#^	1.98 ± 0.33
Time to full weight-bearing (days)	2.38 ± 0.29^#^	3.23 ± 0.32
Total length of hospital stay (days)	5.98 ± 0.89^#^	7.32 ± 0.92

^#^*P* < 0.05 vs. the control group

### Comparison of Hb and Hct levels between the two groups

There was no significant difference in preoperative Hb and Hct levels between the two groups (*P* > 0.05). The levels of Hb and Hct in both groups at POD 1 were lower than those before surgery and were significantly higher in the study group than the control group (*P* < 0.05) (Table 3).

**Table 3:** Comparison of Hb and Hct levels between the two groups ($\overline{x} \pm s$)

Variables	The study group (32)	The control group (32)
Hb (g/L)		
Before surgery	130.25 ± 11.21	131.04 ± 10.24
24 h after surgery	117.24 ± 7.25*^#^	113.16 ± 9.33*^#^
Hct (%)		
Before surgery	40.17 ± 2.87	41.06 ± 2.51
24 h after surgery	36.62 ± 1.77*^#^	32.51 ± 1.56*^#^

*Hb* hemoglobin, *Hct* hematocrit

*P < 0.05 vs. before surgery; ^#^*P* < 0.05 vs. the control group

### Comparison of pain severity at different time points between the two groups

Compared with the control group, the study group had significantly less severe pain at POD 1 and 7 and 1, 3 and 6 months postoperatively (*P* < 0.05) (Table 4).

**Table 4:** Comparison of pain severity at different time points between the two groups ($\overline{x} \pm s$)

Variables	The study group (32)	The control group (32)
POD 1	3.27 ± 0.39^#^	4.34 ± 0.41
POD 7	0.38 ± 0.09^#^	0.87 ± 0.07
1 month postoperatively	0.23 ± 0.07^#^	0.57 ± 0.06
3 months postoperatively	0.18 ± 0.05^#^	0.39 ± 0.05
6 months postoperatively	0.09 ± 0.01^#^	0.21 ± 0.02

*POD* postoperative day

^#^*P* < 0.05 vs. the control group

### Comparison of hip function between the two groups

Compared with the control group, the study group had better hip function at 3, 6 and 12 months postoperatively, and the difference was statistically different (*P* < 0.05) (Table 5).

**Table 5:** Comparison of hip function between the two groups ($\overline{x} \pm s$)

Variables	The study group (32)	The control group (32)
3 months postoperatively	69.83 ± 4.39^#^	65.49 ± 4.87
6 months postoperatively	79.83 ± 5.01^#^	75.49 ± 5.11
12 months postoperatively	89.83 ± 5.87^#^	83.29 ± 5.91

^#^*P* < 0.05 vs. the control group

### Comparison of occurrences of complications between the two groups

All patients were followed up for 12 months. Among them, 1 case of ectopic ossification, Brooker grade I, appeared in both groups 3 months postoperatively. No special treatment was provided as it did not interfere with the mobility of the hip and caused no apparent discomfort in the patient.

**Fig. 3:**
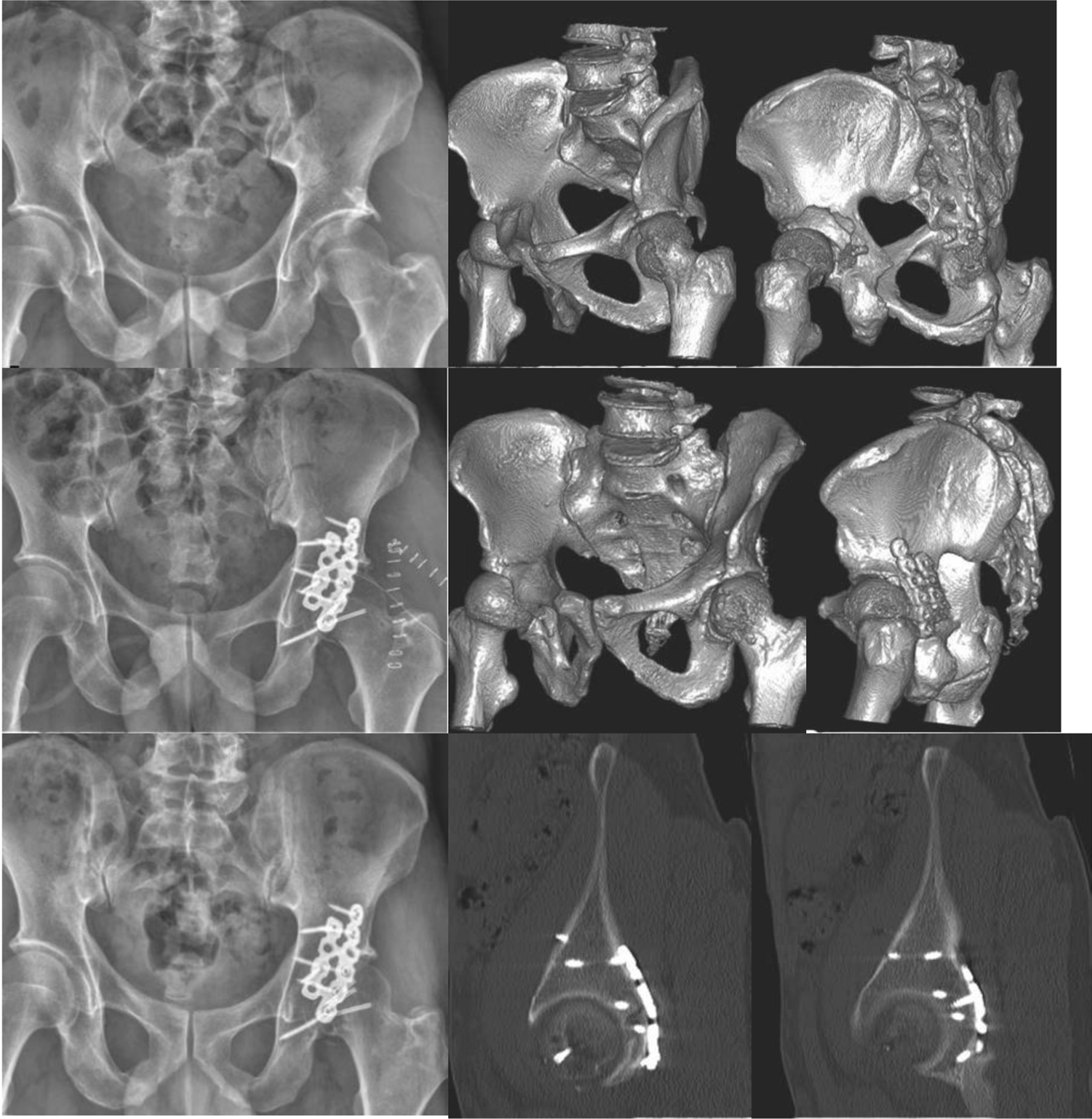
A case of 47-year-old male with traffic accident. All fractures healed well, and no loss of fracture reduction, loosening or fracture of internal fixators occurred during the follow-up

No incision infection, ischemic necrosis of the femoral head, breakage of the internal fixation device, fracture nonunion and loss of fracture reduction and other complications were reported in all patients (Table 6, Fig. 3).

**Table 6:** Comparison of occurrences of complications between the two groups ($\overline{x} \pm s$)

Variables	The study group (32)	The control group (32)
Ectopic ossification	1	1
Surgical incision infection	0	0
Ischemic necrosis of the femoral head	0	0
Breakage of the internal stabilization device	0	0
Fracture nonunion	0	0
Loss of fracture reduction	0	0

^#^*P* < 0.05 vs. the control group

## Discussion

Pipkin divided femoral head fractures into 4 subtypes and classified femoral head fractures combined with acetabulum fracture as Pipkin IV fracture, and its classification mechanism of injury is knee flexion. With the knee in flexion, massive force is conducted via the femur to the femoral head, which severely impacts on the acetabulum and leads to fracture of the femoral head and the acetabulum, often in combination with posterior dislocation of the hip [8]. In young adult patients, open reduction and internal fixation are done [2]. The K–L approach has a wide surgical field and can handle fractures of the posterior acetabulum and the femoral head, which has drawn extensive clinical attention [9, 10]. However, the approach is invasive and is prone to vessel and nerve injury, and can readily lead to complications such as ectopic ossification, and ischemic necrosis of the femoral head. Moreover, it is difficult to carry out reduction and fixation of anteroinferior fractured fragments of the femoral head under direct vision [5]. Therefore, scholars proposed that the anterior S-P approach combined with the K–L approach be used for the treatment of Pipkin IV fracture [11]. Although reduction and fixation of Pipkin IV fracture are effective via the anterior S-P approach combined with the K–L approach patients, it is more invasive and prone to vessel and nerve injury [11]. In 2001, the study by Ganz et al*.* showed that the modified the K–L approach, also called the surgical hip dislocation approach, not only protected the vascular supply of the femoral head, but also could provide reduction and fixation of the exposed fracture site [12]. A previous study [13] has proven that the modified K–L approach for Pipkin IV fracture could yield a satisfactory outcome. However, as the approach is very complicated and invasive, the postoperative incidence of necrosis of the femoral head is as high as 12.5%, while that of ectopic ossification reaches 20%-60% [14].

The direct anterior approach was initially used for tuberculosis drainage in the hip and artificial hip replacement and has been gradually expanded to the treatment of femoral neck and head fractures. In this approach, entry is gained via the superficial internervous plane between the tensor fascia lata and sartorius and via the superficial internervous plane between the gluteus medius and the rectus femoris, which adequately exposes the surgical field. It is minimally invasive and could avoid injuring the lateral femoral cutaneous nerve [15]. The approach is the optimal choice for simple femoral head fractures [16]. The direct posterior approach is modified from the K–L approach, in which the posterior wall acetabular fractures are exposed without transecting the gluteus medius and the lateral rotator muscles and without affecting the trajectory of the medial circumflex femoral artery. It avoids direct contact with the sciatic nerve, and reduction and fixation of the posterior wall acetabular fractures can be done under direct vision, which can achieve a satisfactory outcome in the treatment of posterior wall acetabular fractures [17]. Cao et al*.* [18] used the surgical hip dislocation approach for Pipkin IV fracture with a mean operative time of 165 min and a mean intraoperative estimated blood loss of 850 mL. The study by Bai et al*.* [19] also showed that in the treatment of Pipkin IV fracture by the K–L approach and surgical hip dislocation approach, the surgical hip dislocation approach had a mean operative time of 144.62 min and a mean intraoperative estimated blood loss of 388.46 mL while the K–L approach had a mean operative time of 179.42 min and a mean intraoperative estimated blood loss of 445.8 mL. The current results revealed that the two groups had similar operative time and intraoperative estimated blood loss (*P* > 0.05). Meanwhile, compared with the control group, the study group had a shorter postoperative drainage time, a lower drainage volume, a shorter time to partial and full weight-bearing and a shorter total length of hospital stay (*P* < 0.05) suggesting that the direct anterior approach combined with direct posterior approach in Pipkin IV femoral head fractures does not increase operative time and intraoperative estimated blood loss while shortening postoperative drainage time and reducing drainage volume and shortening time to partial and full weight-bearing and total length of hospital stay, thus promoting rapid recovery of the patients.

In orthopedic surgical patients, postoperative pain may cause spasms of blood vessels and muscles, leading to incision ischemia and causing delay in wound healing, which may impact on the orthopedic surgical outcome of patients [20]. In addition, pain in these patients may lower immunoglobulin levels, reduce immunity and increase the risk of the occurrence of complications. Therefore, the severity of postoperative pain has become an important clinical indicator of surgical outcome. Our results showed that compared with the control group, the study group had less severe pain at POD 1 and 7 and 1, 3 and 6 months postoperatively (*P* < 0.05), suggesting that the direct anterior approach combined with the direct posterior approach for Pipkin IV femoral head fractures may reduce the severity of pain in the patients. Stannaed et al*.* [21] showed that patients treated with the anterior approach had a higher rate of good and satisfactory postoperative hip function than patients treated with the posterior approach; meanwhile, the incidence of ischemic necrosis of the femoral head in patients treated with the posterior approach was three times higher than that of patients treated with the anterior approach, suggesting that the anterior approach is preferred for femoral head fractures. Li et al*.* [22] revealed that the direct anterior approach in artificial femoral head replacement surgery for Alzheimer’s disease patients with femoral neck fracture yielded a good outcome; the approach was less invasive and conducive to hip recovery and had a lower rate of dislocation. Zhang et al*.* [23] also showed that the direct anterior approach in elderly femoral neck fracture patients could more effectively reduce the rate of postoperative complications due to prolonged immobility than the posterolateral approach, lesson postoperative pain, and improve postoperative hip function earlier and more rapidly. Wan et al*.* [24] demonstrated that compared with the posterolateral approach, the direct anterior approach for femoral head replacement in femoral neck fracture had a lower estimated blood loss, shorter hospital stay, more rapid postoperative recovery and shorter time to normal walking and better near term clinical outcomes. Steffen et al*.* [25] showed that the anterior approach had a smaller effect on the femoral head, and the intraoperative position of the affected limb may affect healing. Currently, there are few reports on the direct anterior approach combined with direct posterior approach for Pipkin IV femoral head fractures. Our results revealed that compared with the control group, the study group had better hip function at 3, 6 and 12 months postoperatively (*P* < 0.05), suggesting that the direct anterior approach combined with direct posterior approach for Pipkin IV femoral head fractures is conducive to recovery of hip function. Another study has pointed out that during the operation of femoral neck fracture or femoral head fracture, blood indexes would change due to excessive blood loss [26]. In this study, the levels of Hb and Hct in both groups were monitored before and 24 h after surgery. The results showed that the levels of Hb and Hct in both groups were decreased on POD 1, and the levels of Hb and Hct in the study group were significantly higher than those in the control group (*P* < 0.05), suggesting that the direct anterior approach combined with the direct posterior approach had little influence on blood indexes of patients with Pipkin IV femoral head fractures.

In the direct anterior approach, entry is gained through the superficial internervous plane between the tensor fascia lata and the sartorius, and the deep internervous plane between the gluteus medius and the rectus femoris. After the articular capsule is incised, the dislocated joint of the affected limb is placed under traction and then femoral head fractures are handled. The approach could protect the posterior lateral rotator muscles, prevent injury of the sciatic nerve and lessen the risk of recurrence of hip dislocation. However, the anterior approach is prone to ectopic ossification, while hip dislocation complicated with femoral head fractures is not amenable to reduction via the posterior approach. In the direct posterior approach, the gluteus maximus is bluntly dissected, followed by entry via the plane between the gluteus medius and the piriformis, and the acetabular posterior wall is stripped beneath the periosteum from the posterior column to the ischial spine to expose the posterior acetabular fractures [27]. For posterior wall acetabular fractures, the bone fragments are pulled toward the greater trochanter along the fracture line, and the soft tissues attached to the acetabular posterior wall should not be incised. The vascular supply of fractured bone fragments should be protected, and the posterior hip is stabilized at the same time. In addition, the superior gluteal nerve and vessel bundle that exit from the apex of the incisura ischiadica major should be clearly protected during exposure; meanwhile, the posterior approach has better control of the posterior acetabulum of the femoral head, and better manage hip semi-dislocation, which is difficult to reduce. In addition, the anterior articular capsule does not need to incised, and the vascular supply of the femur is less affected. However, it is a lengthy procedure and has a larger incision size [27]. The current study combined the two approaches and Brooker grade I ectopic ossification developed in one patient each in the two groups 3 months postoperatively. No specific treatment was required as it did not interfere with hip mobility and caused no apparent discomfort. In the study, complications including surgical incision infection, ischemic necrosis of the femoral head, breakage of the internal stabilization device, fracture nonunion and loss of fracture reduction did not occur in any patients. In addition, no patient experienced loss of fracture reduction or loosening or breakage of internal fixation device. The causes may be as follows: (1) All the patients with concurrent hip dislocation underwent emergency closed hip reduction at our hospital or a local hospital, followed by bone traction of the tibial tuberosity or femoral condyle, thereby reducing the occurrences of related complications. Avascular necrosis of the femoral head is an important prognostic predictor of femoral head fracture and is closely related to the injury of blood vessels supplying the femoral head, especially the medial femoral artery. Previous studies [28, 29] showed that the classical K–L approach may cause ischemic necrosis of the femoral head in 5.4% patients, which is as high as 12.5% in the surgical hip dislocation approach. Meanwhile, when posterior wall acetabular fractures are treated by the direct posterior approach, the gluteus medius and the muscles of the lateral rotator group do not need to be transected, and the trajectory of the medial circumflex femoral artery is disturbed, thereby effectively reducing or preventing the incidence of ischemic necrosis of the femoral head. The follow-up period of this study was 12 months, which was shorted than 30 months by Bai et al*.* [19]. Therefore, complications such as avascular necrosis of femoral head may still occur in the later period, and further follow-up is required. (2) A previous study demonstrated that the incidence of postoperative ectopic ossification in femoral head fracture patients reached 6–64% [30]. Current studies consider that when local soft tissues are stimulated by dislocated fracture and surgical trauma, many types of cytokines may together induce the proliferation and differentiation of osteoblast precursors, leading to ectopic ossification. In the current study, entry is gained via planes between muscles, which does not cause excessive injury to the muscles, vessels and soft tissues. Therefore, only 1 patient had ectopic ossification, which was Brooker grade I and did not affect the range of motion of the hip, and no specific treatment was done. In addition, due to the small incision size in the direct anterior approach combined with the direct posterior approach, the view of the surgical field is limited, which requires a high operative skill of the surgeons, especially in obese patients. Excessive traction may injure the sciatic nerve, and occasionally, the incision is lengthened, and the lateral rotator muscles are transected, or conversion is made to other surgical approaches.

In summary, the direct anterior approach combined with the direct posterior approach in Pipkin IV femoral head fractures does not prolong operative time and increase intraoperative estimated blood loss; it can lessen the severity of pain and facilitate recovery of hip function and leads to a favorable prognosis and does not increase the occurrence of complications. However, due to the small sample size, the retrospective nature of the study, and the short duration of follow up, randomized controlled studies in comparison with the direct posterior approach involving a larger population should be conducted in the future.@@@

## Data Availability

The datasets generated and analyzed during the current study are available from the corresponding author on reasonable request.

## Abbreviations

K–L: Kocher–Langenbeck
POD: Postoperative day
